# Oligonucleotide Microarray Analysis of Dietary-Induced Hyperlipidemia Gene Expression Profiles in Miniature Pigs

**DOI:** 10.1371/journal.pone.0037581

**Published:** 2012-05-25

**Authors:** Junko Takahashi, Shiori Waki, Rena Matsumoto, Junji Odake, Takayuki Miyaji, Junichi Tottori, Takehiro Iwanaga, Hitoshi Iwahashi

**Affiliations:** 1 National Metrology Institute of Japan, National Institute of Advanced Industrial Science and Technology, Tsukuba, Ibaraki, Japan; 2 Health Research Institute, National Institute of Advanced Industrial Science and Technology, Takamatsu, Kagawa, Japan; 3 Faculty of Applied Biological Sciences, Gifu University, Gifu, Japan; 4 Japan Farm Co., Ltd, Kagoshima, Japan; The Ohio State Unversity, United States of America

## Abstract

**Background:**

Hyperlipidemia animal models have been established, but complete gene expression profiles of the transition from normal lipid levels have not been obtained. Miniature pigs are useful model animals for gene expression studies on dietary-induced hyperlipidemia because they have a similar anatomy and digestive physiology to humans, and blood samples can be obtained from them repeatedly.

**Methodology:**

Two typical dietary treatments were used for dietary-induced hyperlipidemia models, by using specific pathogen-free (SPF) Clawn miniature pigs. One was a high-fat and high-cholesterol diet (HFCD) and the other was a high-fat, high-cholesterol, and high-sucrose diet (HFCSD). Microarray analyses were conducted from whole blood samples during the dietary period and from white blood cells at the end of the dietary period to evaluate the transition of expression profiles of the two dietary models.

**Principal Findings:**

Variations in whole blood gene expression intensity within the HFCD or the HFCSD group were in the same range as the controls provide with normal diet at all periods. This indicates uniformity of dietary-induced hyperlipidemia for our dietary protocols. Gene ontology- (GO) based functional analyses revealed that characteristics of the common changes between HFCD and HFCSD were involved in inflammatory responses and reproduction. The correlation coefficient between whole blood and white blood cell expression profiles at 27 weeks with the HFCSD diet was significantly lower than that of the control and HFCD diet groups. This may be due to the effects of RNA originating from the tissues and/or organs.

**Conclusions:**

No statistically significant differences in fasting plasma lipids and glucose levels between the HFCD and HFCSD groups were observed. However, blood RNA analyses revealed different characteristics corresponding to the dietary protocols. In this study, whole blood RNA analyses proved to be a useful tool to evaluate transitions in dietary-induced hyperlipidemia gene expression profiles in miniature pigs.

## Introduction

Hyperlipidemia is well recognized as a risk factor for cardiovascular disease (CVD). As diet represents the most important determinant of hyperlipidemia, dietary animal models can be useful for the study of CVD progression [1]. High-fat, high-cholesterol, and high-sugar diets have been shown to induce hyperlipidemia, obesity, and insulin resistance in humans and rodents [2]–[4]. Dietary-induced hyperlipidemia pig models have also been established [5]–[11].

Compared to rodents, pigs are a useful animal model for elucidating the molecular mechanisms underlying the transition from a healthy state to the progression of diseases caused by hyperlipidemia because they are able to breed stably over a long period, and have a similar anatomy and digestive physiology to humans [12], [13]. In addition, miniature pigs are easier to breed and to handle than other non-primates are, making them a convenient species for preclinical tests [14]. In September 2003, the Swine Genome Sequencing Consortium (SGSC) was formed to promote pig genome sequencing under international coordination [15]. The swine research environment has been enhanced since members of the SGSC announced a completed swine genome map in November 2009 [16].

To evaluate temporal changes in gene expression profiles with the progression of dietary-induced alterations, minimally invasive blood sampling, which allows for the direct measurement of immune-responsive blood cells, excels over other invasive biopsy techniques for disease diagnostics and assessment of drug responses, as well as health monitoring. If biomarker candidate genes can be identified from blood analyses, these may be useful for diagnosis in humans. Use of whole blood is preferable to other specimens on two accounts. Firstly, RNA expression and degradation are susceptible to artificial manipulations such as cell separation and extraction. Whole blood manipulation can reduce these risks via the use of RNA blood collection tubes. Secondly, whole blood is an attractive prime tissue due to its critical role in immune responses, metabolism, and communication with cells and the extracellular matrix in almost all body tissues and organs. Whole blood will depart from the normal state when a considerable alteration occurs in some blood cell subpopulations, tissues, or organs. Moreover, blood samples can be obtained repeatedly from miniature pigs, and blood RNA contains an enormous amount of information on the expression of messenger RNA and non-coding functional RNA molecules that are not translated into proteins. Thus, analysis of blood RNA provides an opportunity to detect subtle changes in physiological state. We consider it particularly important to identify gene expression characteristics in whole blood. Microarray techniques allow the detection of genome-wide perturbations in response to different treatments and the measurement of various responses using a multitude of gene probes. Toxicogenomics, in which microarray techniques are specifically used in toxicology tests, has been widely recognized as one of the standard safety procedures for chemicals [17]–[19]. Gene expression microarrays have been used particularly for the screening of genes involved in specific biological processes of interest. Microarrays also allow the clustering of genes according to similar patterns of expression or functions. In this study, we conducted a series of whole blood microarray experiments to evaluate long-term alterations during 27-week feeding periods using specific pathogen-free (SPF) miniature pigs.

There are two main types of dietary protocols for hyperlipidemia pig models, one with cholesterol and animal lipids [5]–[9], and the other with cholesterol, animal lipids, and sucrose [10], [11]. Some studies have focused primarily on a subset of genes, but this approach cannot elucidate whole blood RNA profiles during the process of change. We selected two typical dietary protocols. One was a high-fat and high-cholesterol diet (HFCD) containing 15% lard and 2% cholesterol; the other was a high-fat, high-cholesterol, and high-sucrose diet (HFCSD) containing 15% lard, 2% cholesterol, and 37% sucrose. The present microarray analyses of whole blood were conducted according to the following aspects. The first analysis dealt with similarity among individuals based on the correlation coefficient. Variation among individuals of the same dietary group and between the different dietary periods was examined. The second analysis addressed the function of genes. Up- or down-regulated genes for each dietary protocol were examined by functional categorization. While whole blood RNA derives from white blood cell RNA, whole blood gene expression profiles may not entirely correspond to those of white blood cells [20]. White blood cell microarray analyses conducted at the end of each dietary period are greatly influenced by diet, and the variations between the expression profiles of white blood cells and whole blood were assessed for each dietary group.

## Results

### Characteristics of study subjects

**Figure 1:**
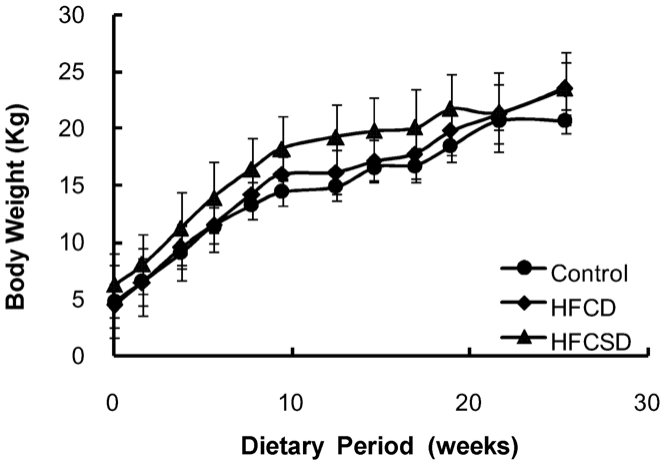
Subject body weights. **•** represents control, **⧫** represents HFCD, and ▴ represents HFCSD. Values correspond to means (SD).

Temporal changes in mean body weights for the 3 dietary groups are shown in Figure 1. One-way ANOVA analysis for dietary-related variation revealed no significant difference at any feeding period except at week 12. In this study, the term “week” refers to the dietary period and not to the period since birth, unless otherwise stated. Table 1 lists the fasting plasma triglyceride concentrations for the group fed the high-fat, high-cholesterol diet (HFCD) and the group fed the high-fat, high-cholesterol, and high-sucrose diet (HFCSD). Almost no changes were observed in fasting plasma triglyceride levels. Fasting plasma total cholesterol concentrations had increased in the HFCD group and the HFCSD group by week 5 of the feeding period (P<0.001) and were maintained between 350 and 1150 mg/dL from weeks 10–27 (Table 2). Fasting plasma high-density lipoprotein cholesterol (HDL-C) concentrations increased and showed significant differences (P<0.001) from weeks 10–27 between two dietary treatment groups and control (Table 3). Fasting plasma low-density lipoprotein cholesterol (LDL-C) concentrations also increased and showed significant differences from weeks 5–27 between two dietary treatment groups and control (Table 4). Fasting plasma glucose concentrations remained unchanged (Table 5). The number of white blood cells and the ratios of granulocytes (basophiles, eosinophils, neutrophils, lymphocytes, and monocytes) to white blood cells were not statistically significant among the three test groups (Table 6–11). The liver (P<0.001) and spleen (P<0.01) weights were increased significantly compared to the controls in both the HFCD and HFCSD groups. In contrast, the heart, kidney, and stomach weights remained unchanged (Table 12).

**Table 1:** Fasting plasma triglyceride levels (mg/dL).

Time point (weeks)	Control	HFCD	HFCSD	*P* †
0	44.5±16.9	42.4±14.4	37.8±21.4	NS
5	25.3±7.2	22.0±22.0	19.5±9.3	NS
10	21.0±2.3	30.4±18.9	30.2±14.7	NS
14	10.8±5.6	7.4±5.0	6.0±3.2	NS
19	10.2±3.6	14.2±14.0	15.6±7.2	NS
23	16.8±7.6	8.2±3.6	8.6±3.2	<.05
27	8.0±2.3	7.2±3.4	12.0±13.5	NS

Values are mean ± SD. NS; not significant.

† *P* values were calculated using a one-way factorial ANOVA.

**Table 2:** Fasting plasma total cholesterol levels (mg/dL).

Time point (weeks)	Control	HFCD	HFCSD	*P* †
0	99.0±21.3	117.4±22.2	100.4±22.5	NS
5	103.8±4.8	620.0±292.6	605.0±131.2	<.001
10	81.2±11.1	780.0±239.3	619.0±205.3	<.001
14	54.4±12.4	646.7±141.4	480.3±43.3	<.001
19	54.6±11.9	745.4±172.7	874.6±208.4	<.001
23	54.2±16.9	562.6±144.9	654.4±219.2	<.001
27	53.0±8.8	541.4±148.5	689.4±267.3	<.001

Values are mean ± SD. NS; not significant.

† *P* values were calculated using a one-way factorial ANOVA.

**Table 3:** Fasting plasma HDL cholesterol levels (mg/dL).

Time point (weeks)	Control	HFCD	HFCSD	*P* †
0	39.3±11.8	50.3±14.8	41.9±11.5	NS
5	50.3±3.1	81.1±6.3	70.7±19.8	NS
10	41.1±5.9	105.2±30.2	96.2±19.6	<.001
14	36.2±5.4	99.3±21.4	106.3±14.9	<.001
19	30.6±7.6	100.3±22.7	117.9±19.8	<.001
23	31.4±8.7	122.3±8.2	110.7±14.2	<.001
27	29.1±4.5	119.2±12.1	106.6±14.6	<.001

Values are mean ± SD. NS; not significant.

† *P* values were calculated using a one-way factorial ANOVA.

**Table 4:** Fasting plasma LDL cholesterol levels (mg/dL).

Time point (weeks)	Control	HFCD	HFCSD	*P* †
0	56.8±11.1	68.4±20.6	56.4±12.9	NS
5	54.0±5.0	318.8±141.7	283.8±49.5	<.01
10	39.2±7.5	339.2±146.5	259.2±107.5	<.01
14	21.8±5.6	212.0±138.4	152.3±43.3	<.05
19	22.8±6.3	236.4±102.7	248.2±78.6	<.001
23	20.8±9.4	220.6±102.2	186.8±46.7	<.001
27	20.6±5.8	201.4±85.2	193.4±86.2	<.01

Values are mean ± SD. NS; not significant.

† *P* values were calculated using a one-way factorial ANOVA.

**Table 5:** Fasting plasma glucose levels (mg/dL).

Time point (weeks)	Control	HFCD	HFCSD	*P* †
0	111.8±12.0	122.6±48.5	119.2±21.9	NS
5	98.6±17.2	100.5±12.0	100.0±23.2	NS
10	93.8±15.7	91.8±27.0	83.0±11.1	NS
14	116.4±32.2	104.6±15.6	108.6±30.5	NS
19	92.4±10.5	95.0±13.1	88.8±25.5	NS
23	87.6±19.8	77.2±7.2	92.0±19.2	NS
27	81.6±7.7	89.6±14.6	101.0±13.0	NS

Values are mean ± SD. NS; not significant.

† *P* values were calculated using a one-way factorial ANOVA.

**Table 6:** White blood cell count (10^2^/µL).

Time point (weeks)	Control	HFCD	HFCSD	*P* †
0	45.8±11.4	61.8±17.1	65.0±16.3	NS
5	68.6±15.5	85.7±14.6	90.0±19.9	NS
10	67.6±12.5	70.8±23.0	78.4±11.1	NS
14	69.4±5.2	87.2±25.9	85.2±13.3	NS
19	68.0±14.8	69.6±12.4	83.4±13.7	NS
23	86.0±21.6	90.2±32.0	100.6±26.1	NS
27	61.8±15.2	75.8±18.6	62.6±34.4	NS

Values are the mean ± SD. NS; not significant.

† *P* values were calculated using a one-way factorial ANOVA.

**Table 7 pone-0037581-t007:** The ratio of basophils to white blood cells (%).

Time point (weeks)	Control	HFCD	HFCSD	*P* †
0	0.0±0.0	0.3±0.5	0.0±0.0	NS
5	0.2±0.4	0.0±0.0	0.2±0.4	NS
10	0.3±0.4	0.2±0.4	0.3±0.4	NS
14	0.2±0.4	0.0±0.0	0.4±0.5	NS
19	0.0±0.0	0.0±0.0	0.0±0.0	NS
23	0.2±0.4	0.0±0.0	0.0±0.0	NS
27	0.1±0.2	0.5±0.5	0.4±0.5	NS

Values are the mean ± SD. NS; not significant.

† *P* values were calculated using a one-way factorial ANOVA.

**Table 8 pone-0037581-t008:** The ratio of eosinophils to white blood cells (%).

Time point (weeks)	Control	HFCD	HFCSD	*P* †
0	2.8±2.6	2.8±1.5	2.0±2.2	NS
5	3.6±1.9	3.0±1.0	3.0±1.4	NS
10	3.1±1.4	3.2±1.3	4.1±1.7	NS
14	3.0±1.9	3.3±1.3	2.4±1.7	NS
19	5.0±2.7	4.1±0.7	5.6±1.8	NS
23	4.6±1.7	6.2±2.7	4.4±0.9	NS
27	2.8±0.8	3.1±1.9	3.4±1.1	NS

Values are the mean ± SD. NS; not significant.

† *P* values were calculated using a one-way factorial ANOVA.

**Table 9 pone-0037581-t009:** The ratio of neutrophils to white blood cells (%).

Time point (weeks)	Control	HFCD	HFCSD	*P* †
0	52.8±16.0	59.6±1.8	54.0±15.5	NS
5	53.8±9.8	55.0±1.0	60.2±3.8	NS
10	43.1±10.3	41.2±6.3	45.5±2.6	NS
14	44.8±7.4	53.0±11.9	51.4±6.1	NS
19	52.2±7.0	48.1±5.4	44.8±4.1	NS
23	56.2±9.2	51.6±3.0	50.6±9.9	NS
27	58.8±13.8	60.1±4.4	48.0±18.5	NS

Values are the mean ± SD. NS; not significant.

† *P* values were calculated using a one-way factorial ANOVA.

**Table 10 pone-0037581-t010:** The ratio of lymphocytes to white blood cells (%).

Time point (weeks)	Control	HFCD	HFCSD	*P* †
0	37.5±11.0	31.1±4.0	36.8±13.1	NS
5	34.8±10.5	36.3±3.5	30.4±2.9	NS
10	45.2±7.4	45.8±6.0	44.7±3.0	NS
14	44.6±9.3	36.2±10.7	39.2±6.4	NS
19	36.9±6.9	42.0±4.9	43.4±3.8	NS
23	33.6±7.6	34.0±2.9	39.6±10.2	NS
27	32.3±13.5	29.5±5.0	41.2±19,5	NS

Values are the mean ± SD. NS; not significant.

† *P* values were calculated using a one-way factorial ANOVA.

**Table 11:** The ratio of monocytes to white blood cells (%).

Time point (weeks)	Control	HFCD	HFCSD	*P* †
0	7.0±3.4	6.3±1.5	7.3±3.9	NS
5	7.6±3.1	5.7±2.5	6.2±1.8	NS
10	8.0±3.2	9.6±1.8	5.4±2.1	NS
14	7.4±1.5	7.5±0.9	6.6±2.3	NS
19	6.0±2.1	5.8±1.1	6.2±1.8	NS
23	5.4±1.3	8.2±1.9	5.2±2.1	N <.05
27	6.0±1.9	6.8±1.0	7.0±1.2	NS

Values are mean ± SD. NS; not significant.

† *P* values were calculated using a one-way factorial ANOVA.

**Table 12:** Effect of diet on organ weight of miniature pigs (g).

Organ	Control	HFCD	HFCSD	*P* †
Heart	149.7±5.5	163.3±4.2	153.7±22.9	NS
Liver	328.0±33.2	667.7±80.9	682.3±21.6	<.001
Kidney	86.3±7.5	99.0±12.5	97.7±4.9	NS
Stomach	169.3±15.2	181.7±13.8	180.0±26.5	NS
Spleen	36.3±3.1	72.0±12.2	74.7±13.3	<.01

Values are mean ± SD. NS; not significant.

† *P* values were calculated using a one-way factorial ANOVA.

### Microarray gene expression profiles – Correlation of gene expression

RNA analyses were conducted on blood samples obtained at weeks 10, 19, and 27 of the feeding periods to characterize the dietary effects on gene expression profiles in whole blood and white blood cells of miniature pigs. Each RNA sample was analyzed by aporcine gene expression microarray consisting of 43603 oligonucleotide probes.

**Figure 2:**
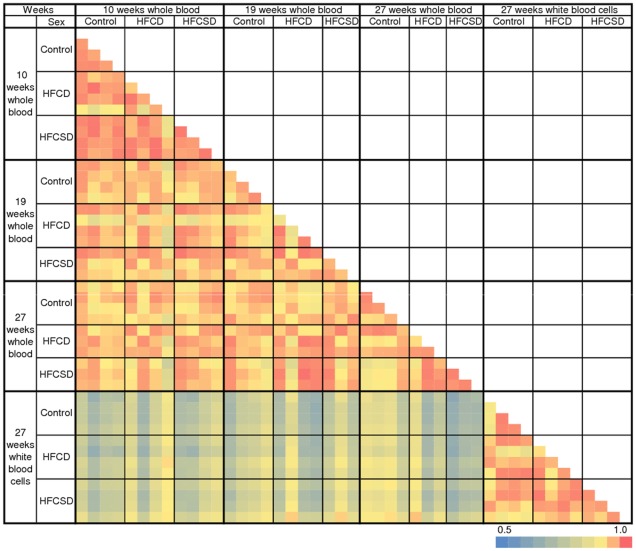
Correlation matrix of dietary-related gene expression profiles of whole blood and white blood cells. This color-coded correlation matrix illustrates pairwise correlations between the levels of gene expression in individuals. Probe sets with normalized signals (log-transformed and scaled) were used to calculate correlations between 45 arrays using Pearson correlation coefficient; signals flagged as “absent” were excluded. The color scale at the bottom indicates the strengths of the correlations.

We evaluated variation in correlation coefficients among individuals on the same diet and between different diet groups. Pearson correlation coefficients were used for the correlation analysis. Correlation coefficients for 45 microarrays in total were obtained for a normalized signals log-scale after excluding “absent” spots, definition of “absent” were described in Materials and Methods. A color-coded pairwise correlation matrix is displayed in Figure 2.

**Figure 3:**
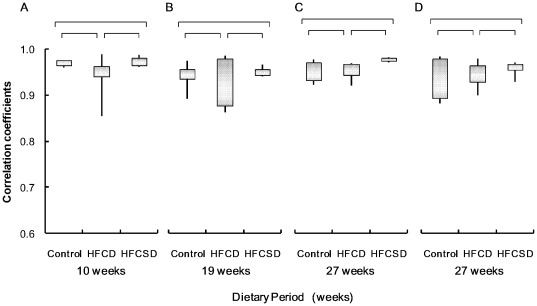
Summary of dietary-related correlation coefficients within the same diet groups. (A) Whole blood after 10 weeks. (B) Whole blood after 19 weeks. (C) Whole blood after 27 weeks. (D) White blood cells after 27 weeks. The bottom and top of the boxes represent the 25th and 75th percentiles respectively. The lower and upper whiskers denote the minimum and maximum values of the data. Using Fisher's Z-transform for normalization the correlation distribution, continuous variables were analyzed by one-way factorial ANOVA followed by Tukey-Kramer multiple comparisons test for multiple groups. Correlations were considered to be statistically significant when ANOVA test among all groups and t-test between 2 groups should p<0.05. NS; not significant.

**Figure 4:**
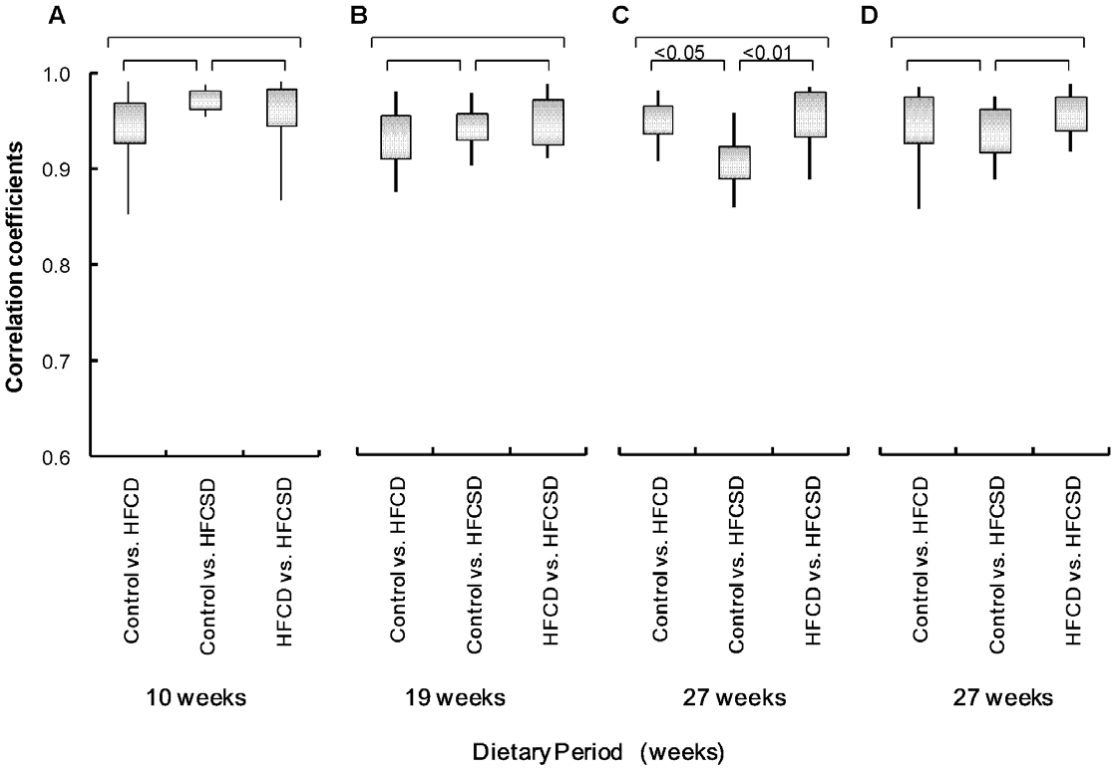
Summary of dietary-related correlation coefficients among different diet groups. (A) Whole blood after 10 weeks. (B) Whole blood after 19 weeks. (C) Whole blood after 27 weeks. (D) White blood cells after 27 weeks. The bottom and top of the boxes represent the 25th and 75th percentiles respectively. The lower and upper whiskers denote the minimum and maximum values of the data. Comparisons of the groups were made with the ANOVA test. NS; not significant.

Figure 3 illustrates the mean correlation coefficients for gene expression profiles among individuals within the same dietary group, showed the individual difference of the gene expression profiles within the dietary groups during dietary period. Figure 4 presents the mean correlation coefficients for gene expression profiles among different diet groups. The correlation coefficients of whole blood expression profiles within the same diet groups were 0.97 (0.01) (mean (standard deviation; SD)), 0.94 (0.05), and 0.97 (0.01) for the control, HFCD, and HFCSD whole blood at 10 weeks, 0.94 (0.03), 0.93 (0.06), and 0.95 (0.01) at 19 weeks, and 0.95 (0.02), 0.95 (0.03), and 0.98 (0.01) at 27 weeks, respectively. The correlation coefficients of white blood cell expression profiles within the same dietary groups were 0.94 (0.05), 0.95 (0.03), and 0.96 (0.02) for the control, HFCD, and HFCSD groups at 27 weeks, respectively. Using Fisher's Z-transformation to normalize the correlation distributions, no significant differences in correlation coefficients among dietary groups were observed at any period during the treatments. This indicates uniformity of dietary-induced hyperlipidemia for our protocols.

Next, we analyzed expression profile correlations among the different diet groups. In Figure 4, “control vs. HFCD” represents the mean correlation coefficient between control and HFCD group individuals. The whole blood correlation coefficients among the different diet groups were 0.95 (0.04), 0.97 (0.01), and 0.96 (0.04) for control vs. HFCD, control vs. HFCSD, and HFCD vs. HFCSD at 10 weeks, 0.93 (0.03), 0.94 (0.02), and 0.95 (0.03) at 19 weeks, and 0.95 (0.03), 0.91 (0.03), and 0.95 (0.03) at 27 weeks, respectively. The white blood cell correlation coefficients among the different diet groups were 0.94 (0.04), 0.94 (0.03), and 0.96 (0.02) for control vs. HFCD, control vs. HFCSD, and HFCD vs. HFCSD at 27 weeks, respectively. Correlations of whole blood expression profiles were statistically significant according to an ANOVA test among all groups at 27 weeks, as a low correlation coefficient was obtained for the control vs. HFCSD groups. This indicates HFCSD differs much from control group and slightly from HFCD 27 weeks in whole blood gene expression profiles.

**Figure 5:**
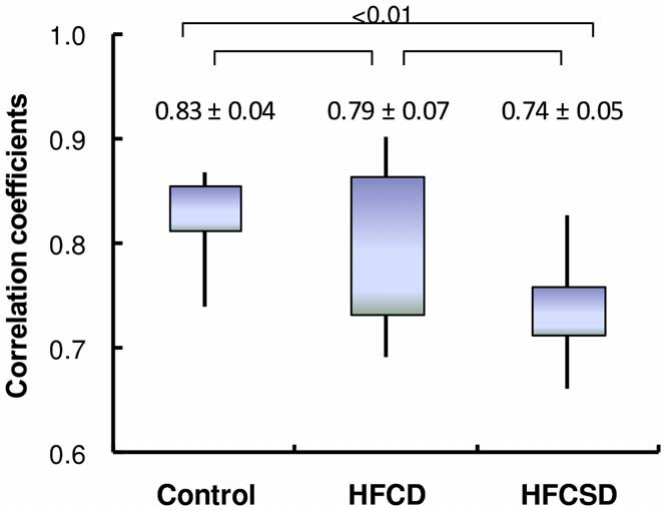
Correlation coefficients between whole blood and white blood cells within the same diet groups. Correlation coefficients were calculated between whole blood and white blood cells within the same diet group at 27 weeks feeding period. The bottom and top of the boxes represent the 25th and 75th percentiles respectively. The lower and upper whiskers denote the minimum and maximum values of the data. Comparisons of the groups were made with the ANOVA test. NS; not significant.

Figure 5 displays the average correlation coefficients between whole blood and white blood cell expression profiles within the same dietary group. The correlation coefficients were 0.83 (0.04), 0.79 (0.07), and 0.74 (0.05) for control, HFCD, and HFCSD at 27 weeks, respectively. Significant differences were observed between the control and HFCSD groups according to an ANOVA analysis using Fisher's Z-transform (P<0.01).

### Assigning known functions to gene expression - Gene ontology annotation

We identified up- and down-regulated genes and classified these according to function using information from the Gene Ontology (GO) Database to understand the observed differences in whole blood gene expression profiles for the different dietary groups. Top-ranked genes with fold changes in expression greater than 2.0 (p<0.05; HFCD, Table 13; HFSCD, Table 14) and less than 0.5 (p<0.05; HFCD, Table 15; HFSCD, Table 16) were selected at 10, 19, and 27 weeks. Genes TC440907, TC448587 (ABCA1), and TC438339 were ranked highest in HFCD and HFCSD during the dietary period. These genes were analyzed using the Database for Annotation, Visualization and Integrated Discovery (DAVID; Table 17, HFCD; Table 18, HFCSD). As a result, the GO categories of many genes up-regulated at the end of the 19-week dietary period in both HFCD and HFCSD groups were related to nucleotide binding (GO: 0000166, GO: GO: 0005524, 0005525, GO: 0017076, GO: 0019001, GO: 00032553, GO: 00032555, GO: 0032561). The GO categories of gene up-regulated after 19 weeks in the HFCD group only were related to catabolic processes (GO: 0009057, GO: 0019941, GO: 0030163, GO: 0043632, GO: 0044257, GO: 0044265,). Many genes down-regulated after 27 weeks in both HFCD and HFCSD groups were in the GO categories related to biological adhesion (GO: 0007155, GO: 0022610). In addition, many genes down-regulated at the end of the 27-week dietary period in the HFCSD group only were related to steroid metabolism and lipid biosynthesis (GO: 0006694, GO: 0008202, GO: 0008203, GO: 0008610, GO: 0016125, GO: 0016126).

**Table 13:** Top 10 significantly up-regulated genes in HFCD.

Whole blood at 10 weeks			Whole blood at 19 weeks			Whole blood at 27 weeks			White blood cells at 27 weeks		
TIGR or Unigene ID	Gene Symbol	Fold Change	TIGR or Unigene ID	Gene Symbol	Fold Change	TIGR or Unigene ID	Gene Symbol	Fold Change	TIGR or Unigene ID	Gene Symbol	Fold Change
TC440907		5.8	TC440907		7.7	TC440907		8.5	TC410790	IRG6	5.9
TC438339		5.7	TC506587		6.2	TC487165	CXCL10	7.3	TC448587	ABCA1	5.3
TC448587	ABCA1	5.3	TC448587	ABCA1	4.8	TC441966	UPK2	5.9	TC440907		5.1
TC414205		3.8	TC407415	MX2	4.7	TC410790	IRG6	5.5	TC447991		4.3
TC407338	MX1	3.4	Ssc.51683	ABCB4	4.3	TC448587	ABCA1	5.3	TC438339		4.0
TC432467		3.1	TC426495		4.3	TC438339		5.1	TC453638		4.0
TC407865		2.9	TC438339		4.0	TC490662	ROMO1	4.7	TC450749		3.5
TC448870		2.9	TC450749		3.9	TC506587		4.4	TC407748	PCD1B	3.4
TC467477	SERPING1	2.7	TC425970	LGALS9	3.8	TC427843		3.6	TC426495		3.3
TC445197		2.6	TC450410		3.8	TC453638		3.6	Ssc.48813		3.2

**Table 14:** Top 10 significantly up-regulated genes in HFCSD.

Whole blood at 10 weeks			Whole blood at 19 weeks			Whole blood at 27 weeks			White blood cells at 27 weeks		
TIGR or Unigene ID	Gene Symbol	Fold Change	TIGR or Unigene ID	Gene Symbol	Fold Change	TIGR or Unigene ID	Gene Symbol	Fold Change	TIGR or Unigene ID	Gene Symbol	Fold Change
TC448587	ABCA1	4.0	TC487165	CXCL10	15.7	NP411727	GRK7	17.9	Ssc.9135		20.1
TC438339		4.0	TC440907		8.4	TC490662	ROMO1	16.9	TC434357		11.6
TC440907		3.8	TC509090		5.1	TC417023		15.6	TC511398		8.5
TC411549		3.1	TC448587	ABCA1	5.0	TC480907		13.5	TC473927		7.1
TC472367		2.4	TC450410		4.9	TC444971		13.3	Ssc.24568		6.7
TC433245		2.1	TC438339		4.5	TC511837		12.7	TC448587	ABCA1	5.7
			TC470851		4.5	TC423285		12.2	TC438339		4.9
			TC480583		4.4	Ssc.46601		11.8	TC440907		4.8
			TC410790	IRG6	4.3	Ssc.56049		11.5	TC430783		4.4
			Ssc.5472		4.1	Ssc.35808		11.2	TC512775		4.3

**Table 15:** Top 10 significantly down-regulated genes in HFCD.

Whole blood at 10 weeks			Whole blood at 19 weeks			Whole blood at 27 weeks			White blood cells at 27 weeks		
TIGR or Unigene ID	Gene Symbol	Fold Change	TIGR or Unigene ID	Gene Symbol	Fold Change	TIGR or Unigene ID	Gene Symbol	Fold Change	TIGR or Unigene ID	Gene Symbol	Fold Change
TC431538		0.2	TC418697		0.1	TC427825		0.2	TC415077		0.2
TC409256		0.2	TC502826		0.1	TC416132		0.2	TC417273		0.2
TC437880		0.2	TC407798	WNT10B	0.2	TC463385		0.2	Ssc.25428		0.3
Ssc.31818		0.2	TC433727		0.2	Ssc.50105		0.3	Ssc.56656	SQLE	0.3
TC434967		0.3	TC495620	IL18	0.2	Ssc.67286		0.3	TC436654		0.4
TC423396		0.3	TC503806		0.2	TC462549		0.3	TC491413		0.4
TC440884		0.3	TC468870		0.2	TC409660		0.3	TC407223	MYO7A	0.4
TC443308		0.3	TC410474		0.2	TC414897	CNN1	0.3	TC478649	PFKFB1	0.4
Ssc.46725		0.3	TC466297	MGP	0.2	TC423618		0.3	TC473047		0.4
Ssc.38146		0.3	TC449799		0.2	TC494014		0.3	TC446195		0.4

**Table 16:** Top 10 significantly down-regulated genes in HFCSD.

Whole blood at 10 weeks			Whole blood at 19 weeks			Whole blood at 27 weeks			White blood cells at 27 weeks		
TIGR or Unigene ID	Gene Symbol	Fold Change	TIGR or Unigene ID	Gene Symbol	Fold Change	TIGR or Unigene ID	Gene Symbol	Fold Change	TIGR or Unigene ID	Gene Symbol	Fold Change
Ssc.56656	SQLE	0.3	TC491388	CCL3L1	0.2	TC442299		0.2	TC491413		0.2
TC426935		0.4	TC407798	WNT10B	0.2	TC407798	WNT10B	0.2	TC414897	CNN1	0.2
Ssc.47297		0.4	TC486323		0.3	Ssc.56656	SQLE	0.2	TC443738		0.2
Ssc.58815		0.4	Ssc.36434		0.3	TC471905		0.3	TC421743		0.2
Ssc.32174		0.4	TC452410	MAOA	0.3	TC429709		0.3	TC412754		0.2
TC452974		0.4	Ssc.56656	SQLE	0.3	TC472114		0.3	TC463385		0.3
TC477452		0.5	TC413430		0.3	TC423826		0.3	TC477521		0.3
TC418062		0.5	TC410474		0.3	TC458674		0.3	NP7655660		0.3
TC491240		0.5	TC509046		0.3	TC417970		0.3	TC442860	TPM1	0.3
TC495442		0.5	Ssc.61167		0.3	TC437991		0.3	TC417854		0.3

**Table 17:** Functional classes of up- or down-regulated genes between HFCD and control.

Weeks	Induction/Repression	Category	Accession	Term	%	P-value
10	Induction	GO MF	GO:0003924	GTPase activity	28.6	1.9e−02
10	Induction	KEGG		complement and coagulation cascades	28.6	2.2e−02
19	Induction	GO MF	GO:0032555	purine ribonucleotide binding	13.6	3.9e−03
19	Induction	GO MF	GO:0032553	ribonucleotide binding	13.6	3.9e−03
19	Induction	GO MF	GO:0000166	nucleotide binding	15.3	4.0e−03
19	Induction	GO MF	GO:0003924	GTPase activity	5.1	4.7e−03
19	Induction	GO MF	GO:0017076	purine nucleotide binding	13.6	6.2e−03
19	Induction	GO BP	GO:0019941	modification-dependent protein catabolic process	5.1	1.9e−02
19	Induction	GO BP	GO:0043632	modification-dependent macromolecule catabolic process	5.1	1.9e−02
19	Induction	GO BP	GO:0044257	cellular protein catabolic process	5.1	2.5e−02
19	Induction	GO BP	GO:0051603	proteolysis involved in cellular protein catabolic process	5.1	2.5e−02
19	Induction	GO MF	GO:0032561	guanyl ribonucleotide binding	6.8	2.6e−02
19	Induction	GO MF	GO:0019001	guanyl nucleotide binding	6.8	2.6e−02
19	Induction	GO MF	GO:0005525	GTP binding	6.8	2.6e−02
19	Induction	GO BP	GO:0030163	protein catabolic process	5.1	2.7e−02
19	Induction	GO BP	GO:0044265	cellular macromolecule catabolic process	5.1	3.5e−02
19	Induction	GO BP	GO:0009057	macromolecule catabolic process	5.1	4.9e−02
27	Induction	KEGG		Toll-like receptor signaling pathway	14.3	1.4e−02
10	Repression	GO MF	GO:0004857	enzyme inhibitor activity	11.1	1.6e−02
10	Repression	GO MF	GO:0004866	endopeptidase inhibitor activity	8.3	4.8e−02
19	Repression	GO BP	GO:0002684	positive regulation of immune system process	7.9	1.3e−02
27	Repression	GO BP	GO:0007155	cell adhesion	11.1	9.6e−03
27	Repression	GO BP	GO:0022610	biological adhesion	11.1	9.6e−03
27	Repression	KEGG		steroid biosynthesis	5.6	4.0e−02

GO MF; GO molecular function, GO BP; GO biological process, Kegg; Kegg pathway.

**Table 18:** Functional classes of up- or down-regulated genes between HFCSD and control.

Weeks	Induction/Repression	Category	Accession	Term	%	P-Value
19	Induction	GO MF	GO:0000166	nucleotide binding	13	3.7e−04
19	Induction	GO MF	GO:0032555	purine ribonucleotide binding	9.8	5.1e−03
19	Induction	GO MF	GO:0032553	ribonucleotide binding	9.8	5.1e−03
19	Induction	GO MF	GO:0017076	purine nucleotide binding	9.8	8.2e−03
19	Induction	GO MF	GO:0005524	ATP binding	6.5	4.8e−02
27	Repression	GO BP	GO:0016125	sterol metabolic process	8.5	4.9e−04
27	Repression	KEGG		steroid biosynthesis	6.4	1.7e−03
27	Repression	GO BP	GO:0008202	steroid metabolic process	8.5	2.3e−03
27	Repression	GO BP	GO:0016126	sterol biosynthetic process	6.4	2.4e−03
27	Repression	KEGG		ECM-receptor interaction	8.5	4.0e−03
27	Repression	GO BP	GO:0008203	cholesterol metabolic process	6.4	9.9e−03
27	Repression	GO BP	GO:0006694	steroid biosynthetic process	6.4	1,1e−02
27	Repression	GO BP	GO:0055114	oxidation reduction	12.8	1.5e−02
27	Repression	GO BP	GO:0007155	cell adhesion	8.5	1.7e−02
27	Repression	GO BP	GO:0022610	biological adhesion	8.5	1.7e−02
27	Repression	GO BP	GO:0008610	lipid biosynthetic process	6.4	4.8e−02

GO MF; GO molecular function, GO BP; GO biological process, Kegg; Kegg pathway.

To investigate potential reasons for the differences in gene expression among the diet groups during the dietary period, Chi-square tests were performed to identify whole blood GO categories for each treatment group vs. the control group. The expected values represented the number of up- and down-regulated genes bearing all GO annotations at each period of the diet, and the observed values represented the number of up- and down-regulated genes bearing each specific GO term. A difference of p<0.05 between groups was considered significant. To identify up- and down-regulated genes, we compared levels of expression for each gene between the control vs. HFCD groups and between the control vs. HFCSD groups at each period using Student's *t*-tests. As the lowest number of genes for which the expectation frequency reached 1 or higher was 140 according to the conditions of observed value, the GO terms, which involve more than 140 genes, were used for the Chi-square tests. The results of the Chi-square tests for up- and down-regulated genes are listed in Tables 19–22. The correlation coefficients of constituent gene between whole blood and white blood cells at 27 weeks were calculated for each GO term.

**Table 19:** Predominant GO terms for which the ratio changed in HFCD and HFCSD.

			Induction/Repressions		Correlation coefficients between whole blood and white blood cells at 27 weeks		
Accession	GO term	Number of genes	HFCD	HFCSD	Control	HFCD	HFCSD
GO:0006954	inflammatory response	155	Repression	Induction/Repression	0.92±0.03	0.97±0.02	0.95±0.02
GO:0000003	reproduction	310	Induction	Induction/Repression	0.91±0.02	0.93±0.03	0.88±0.03

Values are mean ± SD.

**Table 20 pone-0037581-t020:** Predominant GO terms for which the ratio changed in HFCD.

				Correlation coefficients between whole blood and white blood cells at 27 weeks		
Accession	GO term	Number of genes	Induction/Repression	Control	HFCD	HFCSD
GO:0006936	muscle contraction	212	Induction/Repression	0.91±0.03	0.91±0.02	0.91±0.02
GO:0007626	locomotory behavior	207	Induction/Repression	0.88±0.04	0.92±0.05	0.86±0.04
GO:0007517	muscle organ development	199	Repression	0.94±0.05	0.95±0.02	0.94±0.02
GO:0008152	metabolic process	145	Repression	0.93±0.02	0.92±0.05	0.96±0.03

Values are mean ± SD.

**Table 21 pone-0037581-t021:** Predominant GO terms for which the ratio changed in HFCSD.

				Correlation coefficients between whole blood and white blood cells at 27 weeks		
Accession	GO term	Number of genes		Control	HFCD	HFCSD
GO:0006412	Translation	253	Induction/Repression	0.90±0.02	0.92±0.03	0.87±0.03
GO:0009792	embryonic development ending in birth or egg hatching	432	Induction/Repression	0.92±0.02	0.93±0.03	0.90±0.02
GO:0006118	electron transport	200	Induction/Repression	0.82±0.07	0.77±0.09	0.83±0.11
GO:0006366	transcription from RNA polymerase II promoter	209	Induction/Repression	0.96±0.02	0.98±0.01	0.97±0.01
GO:0040010	positive regulation of growth rate	207	Induction	0.92±0.02	0.92±0.04	0.86±0.04
GO:0007166	cell surface receptor linked signaling pathway	185	Repression	0.97±0.01	0.93±0.06	0.97±0.01
GO:0002119	nematode larval development	374	Induction	0.91±0.02	0.93±0.03	0.88±0.03
GO:0006886	intracellular protein transport	191	Induction	0.91±0.06	0.86±0.08	0.86±0.07
GO:0001666	response to hypoxia	189	Repression	0.95±0.02	0.95±0.03	0.92±0.02
GO:0040007	Growth	332	Induction	0.91±0.02	0.93±0.03	0.88±0.03

Values are mean ± SD.

**Table 22:** Predominant GO terms for which the ratio unchanged in HFCD or HFCSD.

			Correlation coefficients between whole blood and white blood cells at 27 weeks		
Accession	GO term	Number of genes	Control	HFCD	HFCSD
GO:0007165	signal transduction	527	0.89±0.04	0.89±0.07	0.80±0.05
GO:0008283	cell proliferation	353	0.91±0.03	0.87±0.07	0.83±0.05
GO:0007267	cell-cell signaling	321	0.94±0.02	0.95±0.03	0.92±0.02
GO:0008285	negative regulation of cell proliferation	288	0.95±0.03	0.87±0.08	0.83±0.06
GO:0006468	protein amino acid phosphorylation	266	0.95±0.01	0.94±0.02	0.90±0.03
GO:0008284	positive regulation of cell proliferation	262	0.92±0.04	0.96±0.02	0.90±0.03
GO:0006916	anti-apoptosis	224	0.91±0.03	0.93±0.03	0.89±0.02
GO:0042493	response to drug	218	0.93±0.03	0.93±0.05	0.85±0.04
GO:0007399	nervous system development	210	0.82±0.06	0.82±0.08	0.74±0.04
GO:0006508	Proteolysis	210	0.90±0.04	0.93±0.03	0.94±0.04
GO:0006470	protein amino acid dephosphorylation	198	0.91±0.07	0.90±0.09	0.84±0.06
GO:0000122	negative regulation of transcription from RNA polymerase II promoter	193	0.93±0.03	0.91±0.04	0.89±0.03
GO:0015031	protein transport	190	0.95±0.03	0.96±0.02	0.94±0.03
GO:0009887	organ morphogenesis	186	0.93±0.03	0.93±0.05	0.92±0.01
GO:0006357	regulation of transcription from RNA polymerase II promoter	179	0.93±0.04	0.94±0.04	0.89±0.03
GO:0001764	neuron migration	174	0.96±0.01	0.95±0.02	0.93±0.01
GO:0007264	small GTPase mediated signal transduction	157	0.97±0.01	0.94±0.05	0.99±0.00
GO:0007275	multicellular organismal development	151	0.95±0.03	0.93±0.04	0.91±0.02
GO:0007420	brain development	150	0.89±0.10	0.89±0.09	0.88±0.09
GO:0043066	negative regulation of apoptosis	145	0.94±0.04	0.95±0.02	0.93±0.01

Values are mean ± SD.

Table 19 lists the GO terms for which significant differences were observed in the HFCD and HFCSD groups relative to the expected values. Inflammatory response elements (GO:0006954) were repressed in the HFCD group, and were both induced and repressed in the HFCSD group. The correlation coefficients between whole blood and white blood cells for expression levels of inflammatory response genes were 0.92 (0.03), 0.97 (0.02), and 0.95 (0.02) for the control, HFCD, and HFCSD groups, respectively. Genes involved in reproduction (GO:0000003) were induced in the HFCD group, and were both induced and repressed in the HFCSD group. The correlation coefficients for expression levels of genes involved in reproduction between whole blood and white blood cells were 0.91 (0.02), 0.93 (0.03), and 0.88 (0.03) for the control, HFCD, and HFCSD groups, respectively.

Table 20 lists the GO terms for which significant differences were observed in the HFCD group compared to the expected values. Muscle contraction (GO:0006936) and locomotor behavior (GO:0007626) elements were both induced and repressed. Muscle organ development (GO:0007517) and metabolic processes (GO:0008152) were repressed.

Table 21 lists the GO terms for which significant differences were observed in the HFCSD group compared to the expected values. Translation (GO:0006412), embryonic development ending in birth or egg hatching (GO:0009792), electron transport (GO:0006118), and transcription from the RNA polymerase II promoter (GO:0006366) elements were both induced and repressed. Positive regulation of growth rates (GO:0040010), nematode larval development (GO:0002119), intracellular protein transport (GO:0006886) and growth (GO:0040007) elements were induced. A cell surface receptor-linked signaling pathway (GO:0007166) and responses to hypoxia (GO:0001666) were repressed.

Table 22 lists the GO terms for which ratios to the expected values were unchanged in the HFCD and HFCSD groups. In addition, the ratios of up- and down-regulated genes to the each observed values were unchanged at 27 weeks.

**Figure 6:**
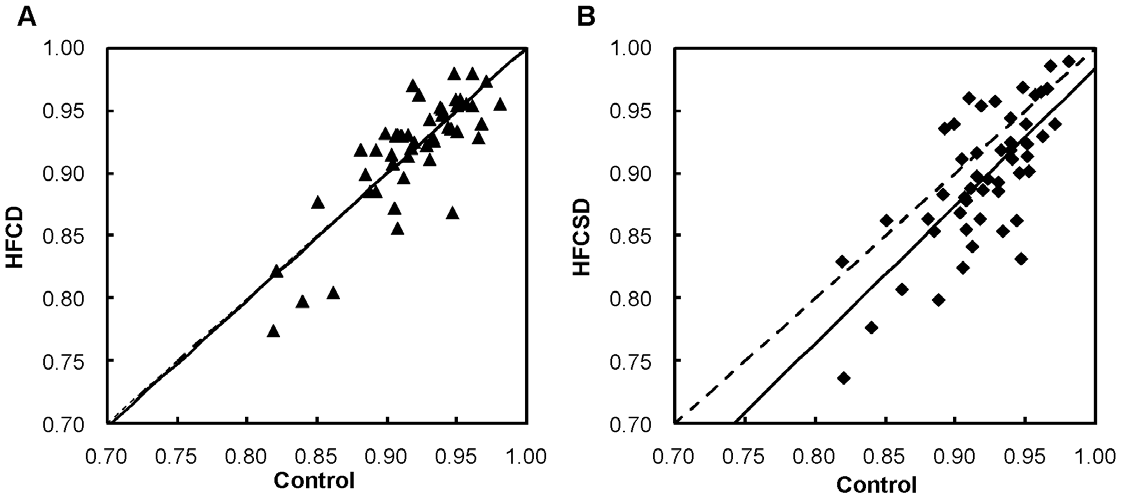
Scatter plot of dietary-related correlation coefficients. (A) Control vs. HFCD. (B) Control vs. HFCSD. The correlation coefficient between whole blood and white blood cells after 27 weeks of each GO tem was plotted for each spot. The solid line represents the regression line. The dashed line represents the slope equal to 1.

Figure 6 depicts a scatter plot of correlation coefficients between whole blood and white blood cells for each GO term, selected for the Chi-square tests, at 27 weeks of each dietary treatment group relative to the control group. The slope of the HFCD to the controls regression line was 1.007 (p<0.001). The slope of the HFCSD to the controls regression line was 1.097 (p<0.001), indicating that the correlation coefficients between whole blood and white blood cell expression levels for many GO terms were low. The predominant GO terms with low correlation coefficients in the HFCSD group were nervous system development (GO:0007399), biological processes (GO:0008150), signal transduction (GO:0007165), regulation of transcription, DNA-dependent (GO:0006355), and cell proliferation (GO:0008283). In contrast, the predominant GO terms with high correlation coefficients in the HFCSD group were skeletal system development (GO:0001501), small GTPase mediated signal transduction (GO:0007264), synaptic transmission (GO:0007268), cell surface receptor linked signaling pathway (GO:0007166), and transcription from the RNA polymerase II promoter (GO:0006366).

**Figure 7:**
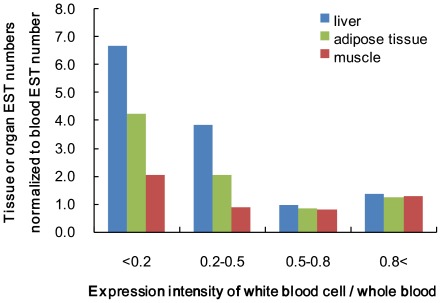
The relation of tissue or organ ESTs to the white blood cell contribution indicator. The X-axis indicates the expression intensity ratio of white blood cells to whole blood for each gene as the white blood cell contribution indicator in our experiments. The Y-axis indicates the liver, adipose tissue, or muscle EST numbers normalized to the blood EST number of each gene in Unigene, an NCBI database of the transcriptome.

The intensity ratio of white blood cells to whole blood is a contribution indicator of the white blood cell RNA to whole blood gene expression. To focus on obesity-related organs, i.e., the liver, adipose tissue, and muscle, the relative numbers of ESTs for these organs to blood ESTs for each gene were calculated using EST profiles from the Unigene NCBI database of the transcriptome. The normalized EST values increase when the contribution indicator is small, as shown in Figure 7.

## Discussion

This study aimed to evaluate the transition of gene expression profiles caused by dietary-induced hyperlipidemia through blood microarray analyses of miniature pigs during a 27-week dietary period.

Dietary-induced hyperlipidemia miniature pig models have previously been established. There are 2 main types of dietary protocol, one containing cholesterol and animal lipids [5]–[9], and the other containing cholesterol, animal lipids, and sucrose [10], [11]. Some studies have focused their attention on certain kinds of candidate genes with specific functions, but this has not clarified a complete projection of whole blood RNA profiles of the transitions caused by diet-induced hyperlipidemia. Excessive exposure to dietary fats and/or sugars is an essential factor in the initiation of obesity and metabolic syndrome-associated pathologies, two typical conditions associated with diet-induced hyperlipidemia. The fasting plasma total cholesterol level increased within a month, and then, either remained high or decreased in the high-fat and high-cholesterol diet (HFCD) models [5], [6], [9]. In contrast, fasting plasma total cholesterol levels increased throughout the dietary period in the high-fat, high-cholesterol, and high-sucrose diet (HFCSD) models [11]. Therefore, in the present study, hyperlipidemia was induced by the administration of a high-fat and high-cholesterol diet or a high-fat, high-cholesterol, and high sucrose diet to Clawn miniature swine.

Fasting plasma lipid values increased rapidly and were maintained at a high level during the 27-week feeding period under both feeding treatments. However, fasting plasma glucose concentrations remained unchanged. The liver and spleen weights increased significantly after the 27 weeks, and fatty livers were reported based on autopsies of individuals from both treatment groups. There was no significant difference in body weight, hematology, or other biochemical aspects of blood between individuals from the 2 dietary treatments.

### Gene expression profiles of dietary-induced hyperlipidemia for whole blood RNA

We used whole blood to evaluate the transition of gene expression profiles. Whole blood RNA is easy to handle compared to isolated white blood cell RNA. In addition, whole blood contains a heterogeneous mixture of subpopulations of blood cells. Associated changes will be reflected on whole blood RNA once a great change has occurred in the composition and expressing condition of subpopulations, tissues, or organs. We previously evaluated the “healthy state” gene expression profile by whole blood microarray analyses of miniature pigs of different age groups, and identified characteristics of age-related gene expression by taking into account the change in the number of expressed genes by age and the similarities of gene expression intensity between individuals [21]. The report on the healthy state of miniature pigs found that the correlation coefficients within the same age groups were 0.87 (0.04), 0.93 (0.03), 0.98 (0.01), and 0.96 (0.02), for the fetal stage, and for 12-, 20-, and 30-week-old male pigs, respectively. Variation in gene expression was greatest for younger subjects and diminished with age. These results indicate that uniformity of laboratory animals can be expected in miniature pigs after 20 weeks of age.

In this study, feeding treatments commenced when the pigs were 12 weeks old, RNA analysis was conducted on whole blood sampled after 10, 19, and 27 weeks of the feeding period, and on white blood cell RNA after 27 weeks. Variation in whole blood gene expression intensity among individuals within either the HFCD or the HFCSD group was in the same range as that of the controls at any period, indicating uniformity of dietary-induced hyperlipidemia expression profiles in miniature pigs.

### Effects of white blood cells on whole blood gene expression profiles in dietary-induced hyperlipidemia

Most of the nucleated cells in blood are white blood cells such as neutrophils, T cells, B cells, and monocytes. Min et al. reported highly correlated results (r^2^ = 0.85) for 8,273 genes expressed in both whole blood RNA and peripheral blood mononuclear cell (PBMCs) RNA samples from healthy volunteers [20]. Other researchers have conducted a large-scale genome-wide expression analysis of white blood cell subpopulations [22]. That study indicated that correlation coefficients for T cells and monocytes among different healthy subjects were 0.98 (0.01) and 0.97 (0.01), respectively. However, the correlation coefficient between T cells and monocytes for the same subjects (n = 5) was 0.88 (0.01), indicating varied correlations between white blood cell subpopulations [22]. We believe that no effects of composition ratio of white blood cell subpopulations were observed in our study, because the ratios of granulocytes (neutrophils, eosinophils, and basophils), lymphocytes, and monocytes to white blood cells were statistically insignificant among the three test groups.

In previous studies, tumor-derived RNA was detected in the circulation of cancer patients [23], [24]. It has also been demonstrated that fetal RNA can be detected in maternal plasma [25]. These results indicate that whole blood RNA may contain RNA originating from the tissues and/or organs. Hyperlipidemia is one of the risk factors associated with atherosclerosis. Atherosclerosis was induced by the administration of a high-fat and high-diet to Göttingen miniature swine for a 6-month period [5]. The liver and spleen weights were increased significantly compared to the controls in both the HFCD and HFCSC groups in our experiment at the end of each dietary period. Thus white blood cell microarray analyses were conducted at the end of each dietary period, as the tissues and/or organs, such as the liver, spleen, and blood vessels, were presumed to be influenced by dietary treatment.

The average white blood cell correlation coefficients within the HFCD and HFCSD groups were in the same range as that of the controls after the 27-week feeding period. However, variation in whole blood gene expression intensity between the HFCSD group and the control group was statistically significant, whilst variation in white blood cell gene expression intensity between the HFCSD group and the control group was not significant after the 27-week feeding treatments. In addition, the HFCSD correlation coefficient between whole blood and white blood cells after 27 weeks was significantly lower than that of the control and HFCD groups.

The intensity ratio of white blood cell gene expression to that of whole blood shows the contribution of white blood cell RNA to whole blood RNA samples. The intensity ratio of white blood cells to whole blood is, therefore, considered as the contribution indicator. We assume that the low intensity ratio of white blood cell to whole blood gene expression indicates a greater contribution of tissues and/or organs RNA to whole blood RNA. We then compared the EST numbers of the tissue or organ with the contribution indicator, focusing on obesity-related organs such as the liver, adipose tissue, and muscle. The number of gene ESTs for each tissue or organ normalized to blood ESTs becomes greater when the contribution indicator is small. As a result, we suggest that RNAs originating from tissues and/or organs are present in whole blood.

### Characteristics of gene expression profiles in dietary-induced hyperlipidemia

It is generally acknowledged that excessive exposure to dietary lipids disrupts the homeostasis of cellular metabolism and triggers an inflammatory response in adipose tissue [26]. An enhanced inflammatory response has been observed in the livers of mice fed on high-fat diets and in skeletal muscles of Otsuka Long-Evans Tokushima Fatty (OLETF) rats using microarrays [27]. We examined dietary-induced transitions of gene expression profiles for genes bearing GO terms. Major changes included an induction of proteins involved in catabolic processes and protein metabolism after a 19-week dietary period, especially in the HFCD group, and a reduced expression of proteins involved in steroid metabolism and lipid biosynthesis after a 27-week dietary period, especially in the HFCSD group.

In whole blood samples, some genes involved in inflammatory responses (GO: 0006954) were down-regulated in the HFCD group, whilst some genes involved in inflammatory responses were up-regulated and others were down-regulated in the HFCSD group.

It has been established that skeletal muscle is an obesity-related organ, such as the liver and adipose tissue, in association with insulin resistance [26], [28], [29]. Indeed, 2 out of 4 GO terms (muscle contraction, GO: 0006936, muscle organ development, GO: 0007517) that were statistically significant in the HFCD group were related to muscle function. Genes involved in reproduction (GO: 000003) were induced in the HFCD group, and were either induced or repressed in the HFCSD group. Asexual reproduction is the process by which an organism creates a genetically similar or identical copy of itself without the contribution of genetic material from another individual, and some genes involved in asexual reproduction are linked to the repair of damaged organs. Genes involved in translation (GO: 0006412), positive regulation of growth rate (GO: 0040010), and growth (GO: 004007) were induced in the HFCSD group, and these processes are also linked to organ repair. Meanwhile, GO terms that were statistically significant in the HFCSD group were mainly associated with cellular volatility, such as cellular activity, cell growth, or cellular responses.

We examined correlations between whole blood and white blood cells for genes bearing GO terms. The correlation coefficients for each GO term were calculated for the control, HFCD, and HFCSD groups after the 27-week feeding treatments. As a result, GO terms related to white blood cell function, including inflammatory responses (GO: 0006954), and cell surface receptor-linked signaling pathways (GO: 0007166) show high correlation coefficients in the control and dietary groups. In contrast, GO terms related to the repair of damaged organs, including translation (GO: 0006412), positive regulation of growth rate (GO: 0040010), and growth (GO: 004007), show low correlation coefficients in the HFCSC group.

The differences in the scatter plot regression slopes between the HFCD and control treatments and between the HFCSD and control treatments did not indicate a decrease in the extraction efficiency of RNA due to inhibitory substances in blood. In a previous study of microarray cDNA expression profiles using 23 healthy porcine tissue specimens, a large portion of the genes exhibited tissue-specific expression in agreement with mappings to gene descriptions [30]. In our study, the minimum correlation coefficient for each GO term was 0.737 (0.038), while the maximum was 0.989 (0.004), indicating different values related to functions. The reason for the lower correlation may be due to the differences in gene expression between blood cells and organs, and because a stronger tendency for a decrease in correlation strength was observed in the HFCSD group as compared to the HFCD group. Our EST profile analysis also supported this assumption.

Statistically significant differences in fasting plasma lipids and glucose levels between the HFCD and HFCSD groups were not observed. However, blood RNA analyses demonstrated differences in the characteristics of dietary components between these groups. By considering variation in the dietary-induced hyperlipidemia gene expression profiles of miniature pigs, we have established that whole blood RNA analyses can be used in practical applications. The blood RNA diagnostics under development may eventually be useful for monitoring human health.

## Materials and Methods

### Animals

Fifteen 12-week-old, male Clawn miniature pigs were housed individually in cages of 1.5 m^2^ at the breeder's specific pathogen-free (SPF) facility (Japan Farm Co., Ltd, Kagoshima, Japan) for 27 weeks. Body weights at the beginning of the experiment were 5.1 (2.6) kg (mean (standard deviation; SD)). During this period, 5 pigs were fed with 450 g/day standard dry feed (Kodakara73, Marubeni Nisshin Feed Co., Ltd., Tokyo Japan), and had unlimited access to water (control group). Five pigs were fed a high-fat, high-cholesterol diet containing 15% lard and 2% cholesterol (HFCD group). The 5 remaining pigs were fed a high-fat, high-cholesterol and high-sucrose diet containing 15% lard, 2% cholesterol, and 37% sucrose (HFCSD group). During dissections, the heart, liver, kidney, stomach, and spleen were excised and weighed immediately.

### Hematology and clinical chemistries

Blood samples were collected from the superior vena cava after 5, 10, 14, 19, 23, and 27 weeks of the feeding period. Blood (EDTA), plasma (EDTA), and serum samples for hematology and biochemical tests were collected 24 hours after fasting. Hematology and biochemical tests were conducted by Clinical Pathology Laboratory, Inc. (http://www.patho.co.jp/index.html) (Kagoshima, Japan) using standard clinical methods.

### MIAME compliance and data availability

The microarray experiments described in this manuscript were MIAME compliant and the raw data have been deposited in the Gene Expression Omnibus (GEO) database (Accession number GSE 32616, http://www.ncbi.nlm.nih.gov/geo/query/acc.cgi?acc=GSE32616.

### Preparation of samples and microarray assays

Whole blood samples for microarray analyses were collected from each subject in PAXgene™ tubes (Qiagen/BD GmbH, UK), incubated at room temperature for 4 hours for RNA stabilization, and then stored at −80°C. RNA was extracted from whole blood using the PAXgene™ Blood RNA System Kit (Qiagen GmbH, Germany) according to the manufacturer's guidelines. RNA from white blood cells was extracted from whole blood samples using a LeukoLOCK Total RNA Isolation kit (Ambion, Austin, TX). Isolations were performed according to the manufacturer's protocol. The quality of the purified RNA was verified using an Agilent® 2100 Bioanalyzer (Agilent Technologies, Santa Clara, CA). RNA concentrations were determined using a NanoDrop® ND-1000 spectrophotometer (NanoDrop Technologies, Wilmington, DE). Fluorescent cyanine 3-CTP–labeled cRNA was used for hybridization onto porcine oligo microarray slides (#G2519F#20109, Agilent Technologies) containing 43,603 oligonucleotide probes at 65°C for 17 h. The hybridized microarray slides were washed according to the manufacturer's instructions and were scanned with an Agilent DNA Microarray Scanner (#G2565BA, Agilent Technologies) at 5-µm resolution. The scanned images were analyzed numerically using Agilent Feature Extraction Software version 9.5.3.1. (Agilent Technologies).

### Microarray data analysis

Normalized data using quantile normalization were analyzed using GeneSpring GX software version 10.0.1 (Agilent Technologies). The Gene Ontology (GO) Database (http://www.geneontology.org/) was used to categorize gene expression profiles functionally. GO terms were obtained from the TIGR pig gene indices, Porcine version 14.0 3-11-10 (http://compbio.dfci.harvard.edu/cgi-bin/tgi/gimain.pl?gudb=pig). The TC Annotator List includes the gene number and the GO terms. Out of the 43,603 probes used in the Agilent porcine microarray (#G2519F#20109), GO annotations were available for 6,019 genes. Microarray cDNA probes were classified according to GO terms for different biological processes.

For the microarray data analyses, we focused particularly on the variation of dietary-related gene expression profiles. Initially, microarray spots of interest were divided into 2 groups: “absent” and “present,” using the flag values provided by the scanner. Background levels were determined from the spots outside of the gene probing area. “Absent” was assigned to spots with a signal intensity that was less than that of the background level, while the rest were marked “present.” Only data for “present” spots were used for the analyses.

The intensity ratio of white blood cell gene expression to that of whole blood is a contribution indicator for white blood cell RNA to whole blood RNA. The relation of tissues or organs ESTs to the white blood cell contribution indicator was examined. To focus on obesity-related organs, i.e., the liver, adipose tissue, and muscle, the relative EST numbers of these organs to blood ESTs for each gene were calculated using EST profiles from the Unigene NCBI database of the transcriptome. An EST profile breakdown of 22,000 porcine genes by body site is available, comprising 40 organ types, such as the lung, ovary, liver, adipose tissue, muscle, and blood. The profiles show gene expression patterns inferred from EST counts and cDNA library sources (http://www.ncbi.nlm.nih.gov/UniGene/).

### Statistical analysis

Continuous variables were analyzed using a 1-way factorial ANOVA followed by a Tukey-Kramer multiple comparisons test for multiple groups. After excluding the unexpressed genes from each set of array data, Pearson correlation coefficients were calculated to identify similarities in gene expression among individuals. Pearson correlation coefficients were analyzed by a 1-way factorial ANOVA using Fisher's Z-transform to normalize the correlation distribution.

Correlations were considered statistically significant for ANOVA tests among all groups and *t*-tests between 2 groups when p<0.05. All values were expressed as non-transformed mean (standard deviation (SD)). Genes with a fold change greater than 2.0 (p<0.05) and less than 0.5 (p<0.05) after 10, 19, and 27 weeks were identified. These genes were mapped to the Gene Ontology and KEGG pathway in the Database for Annotation, Visualization and Integrated Discovery (DAVID Bioinformatics Resources 6.7, National Institute of Allergy and Infectious Diseases, http://david.abcc.ncifcrf.gov/) [31], [32]. Chi-square tests were performed for feature extractions of GO terms. The expected values were the number of up- and down-regulated genes bearing all GO annotations, and the observed values were specific to each GO term. Simple linear regressions were performed for the scatter plots to obtain the slopes and intercepts, and the significance of each regression slope was verified.

### Ethical considerations

All experimental protocols were approved by the Committee for the Care and Use of Experimental Animals at AIST (Permit Number: 2009-055A).

## References

[1] Lissner L, Heitmann BL (1995). Dietary fat and obesity: evidence from epidemiology.. Eur J Clin Nutr.

[2] Radonjic M, de Haan JR, van Erk MJ, van Dijk KW, van den Berg SAA (2009). Genome-wide mRNA expression analysis of hepatic adaptation to high-fat diets reveals switch from an inflammatory to steatotic transcriptional program.. PLoS ONE.

[3] Russell JC, Proctor SD (2006). Small animal models of cardiovascular disease: tools for the study of the roles of metabolic syndrome, dyslipidemia, and atherosclerosis.. Cardiovasc Pathol.

[4] Oron-Herman M, Kamari Y, Grossman E, Yeger G, Peleg E (2008). Metabolic syndrome: comparison of the two commonly used animal models.. Am J Hypertens.

[5] Kobari Y, Koto M, Tanigawa M (1991). Regression of diet-induced atherosclerosis in Güttingen Miniature Swine.. Lab Anim.

[6] De Keyzer D, Karabina SA, Wei W, Geeraert B, Stengel D (2009). Increased PAFAH and oxidized lipids are associated with inflammation and atherosclerosis in hypercholesterolemic pigs.. Arterioscler Thromb Vasc Biol.

[7] Orbe J, Rodriguez JA, Calvo A, Grau A, Belzunce MS (2001). Vitamins C and E attenuate plasminogen activator inhibitor-1 (PAI-1) expression in a hypercholesterolemic porcine model of angioplasty.. Cardiovasc Res.

[8] de Smet BJ, Kuntz RE, van der Helm YJ, Pasterkamp G, Borst C (1997). Relationship between plaque mass and neointimal hyperplasia after stent placement in Yucatan micropigs.. Radiology.

[9] Bowles DK, Heaps CL, Turk JR, Maddali KK, Price EM (2004). Hypercholesterolemia inhibits L-type calcium current in coronary macro-, not microcirculation.. J Appl Physiol.

[10] Yin W, Liao D, Kusunoki M, Xi S, Tsutsumi K (2004). NO-1886 decreases ectopic lipid deposition and protects cells in diet-induced diabetic swine pancreatic.. J Endocrinol.

[11] Zhang C, Yin W, Liao D, Huang L, Tang C (2006). NO-1886 upregulates ATP binding cassette transporter A1 and inhibits diet-induced atherosclerosis in Chinese Bama minipigs.. J Lipid Res.

[12] Lunney JK (2000). Advances in swine biomedical model genomics.. Int J Biol Sci.

[13] Simon GA, Maibach HI (2000). The pig as an experimental animal model of percutaneous permeation in man: qualitative and quantitative observations – an overview.. Skin Pharmacol Appl Skin Physiol.

[14] Vodicka P, Smetana K, Dvoránková B, Emerick T, Xu YZ (2005). The miniature pig as an animal model in biomedical research.. Ann N Y Acad Sci.

[15] Schook LB, Beever JE, Rogers J, Humphray S, Archibald A (2005). Swine Genome Sequencing Consortium (SGSC): a strategic roadmap for sequencing the pig genome.. Comp Funct Genomics.

[16] Archibald AL, Bolund L, Churcher C, Fredholm M, Groenen MA (2010). Pig genome sequence–analysis and publication strategy.. BMC Genomics.

[17] Williams-Devane CR, Wolf MA, Richard AM (2009). Toward a public toxicogenomics capability for supporting predictive toxicology: survey of current resources and chemical indexing of experiments in GEO and ArrayExpress.. Toxicol Sci.

[18] Pennie W, Pettit SD, Lord PG (2004). Toxicogenomics in risk assessment: an overview of an HESI collaborative research program.. Environ Health Perspect.

[19] Tong W, Cao X, Harris S, Sun H, Fang H (2003). ArrayTrack–supporting toxicogenomic research at the U.S. Food and Drug Administration National Center for Toxicological Research.. Environ Health Perspect.

[20] Min JL, Barrett A, Watts T, Pettersson FH, Lockstone HE (2010). Variability of gene expression profiles in human blood and lymphoblastoid cell lines.. BMC Genomics.

[21] Takahashi J, Misawa M, Iwahashi I (2011). Oligonucleotide microarray analysis of age-related gene expression profiles in miniature pigs.. PLoS ONE.

[22] Cobb JP, Mindrinos MN, Miller-Graziano C, Calvano SE, Baker HV (2005). Application of genome-wide expression analysis to human health and disease.. Proc Natl Acad Sci USA.

[23] Kopreski MS, Benko FA, Kwak LW, Gocke CD (1999). Detection of tumor messenger RNA in the serum of patients with malignant melanoma.. Clin Cancer Res.

[24] Lo KW, Lo YMD, Leung SF, Tsang YS, Chan LY (1999). Analysis of cell-free Epstein-Barr virus associated RNA in the plasma of patients with nasopharyngeal carcinoma.. Clin Chem.

[25] Poon LL, Leung TN, Lau TK, Lo YM (2000). Presence of fetal RNA in maternal plasma.. Clin Chem.

[26] Hotamisligil GS (2006). Inflammation and metabolic disorders.. Nature.

[27] Hayashi Y, Kajimoto K, Iida S, Sato Y, Mizufune S (2010). DNA microarray analysis of whole blood cells and insulin-sensitive tissues reveals the usefulness of blood RNA profiling as a source of markers for predicting type 2 diabetes.. Biol Pharm Bull.

[28] Roberts CK, Barnard RJ, Liang KH, Vaziri ND (2002). Effect of diet on adipose tissue and skeletal muscle VLDL receptor and LPL: implications for obesity and hyperlipidemia.. Atherosclerosis.

[29] Krotkiewski M (1994). Role of muscle morphology in the development of insulin resistance and metabolic syndrome.. Presse Med.

[30] Hornshøj H, Conley LN, Hedegaard J, Sørensen P, Panitz F (2007). Microarray expression profiles of 20.000 genes across 23 healthy porcine tissues.. PLoS One.

[31] Huang da W, Sherman BT, Lempicki RA (2009). Bioinformatics enrichment tools: paths toward the comprehensive functional analysis of large gene lists.. Nucleic Acids Res.

[32] Dennis G, Sherman BT, Hosack DA, Yang J, Gao W (2003). DAVID: Database for Annotation, Visualization, and Integrated Discovery.. Genome Biol.

